# Zonal characterization and differential trilineage potentials of equine intrasynovial deep digital flexor tendon-derived cells

**DOI:** 10.1186/s12917-021-02793-1

**Published:** 2021-04-01

**Authors:** Vivian G. Quam, Nadine N. Altmann, Matthew T. Brokken, Sushmitha S. Durgam

**Affiliations:** Department of Veterinary Clinical Sciences, College of Veterinary Medicine, The Ohio State University, 601 Vernon L. Tharp Street, Columbus, OH USA

**Keywords:** Intrasynovial deep digital flexor tendon, Tendon-derived cells, Trilineage differentiation, Chondrogenic phenotype

## Abstract

**Background:**

Intrasynovial deep digital flexor tendon (DDFT) injuries occur frequently and are often implicated in cases of navicular disease with poor outcomes and reinjuries. Cell-based approaches to tendon healing are gaining traction in veterinary medicine and ultimately may contribute to improved DDFT healing in horses. However, a better understanding of the innate cellular characteristics of equine DDFT is necessary for developing improved therapeutic strategies. Additionally, fibrocartilaginous, intrasynovial tendons like the DDFT are common sites of injury and share a poor prognosis across species, offering translational applications of this research. The objective of this study is to isolate and characterize tendon-derived cells (TDC) from intrasynovial DDFT harvested from within the equine forelimb podotrochlear bursa. TDC from the fibrocartilaginous and tendinous zones are separately isolated and assessed. Flow cytometry is performed for mesenchymal stem cell (MSC) surface markers (CD 29, CD 44, CD 90). Basal tenogenic, osteogenic and chondrogenic markers are assessed via quantitative real time-PCR, and standard trilineage differentiation is performed with third passage TDC from the fibrocartilaginous (fTDC) and tendinous (tTDC) zones of DDFT.

**Results:**

Low-density plating isolated homogenous TDC populations from both zones. During monolayer passage, both TDC subpopulations exhibited clonogenicity, high in vitro proliferation rate, and fibroblast-like morphology. fTDC and tTDC were positive for MSC surface markers CD90 and CD29 and negative for CD44. There were no significant differences in basal tenogenic, osteogenic or chondrogenic marker expression between zones. While fTDC were largely restricted to chondrogenic differentiation, tTDC underwent osteogenic and chondrogenic differentiation. Both TDC subpopulations displayed weak adipogenic differentiation potentials.

**Conclusions:**

TDC at the level of the podotrochlear bursa, that potentially could be targeted for enhancing DDFT injury healing in horses were identified and characterized. Pending further investigation, promoting chondrogenic properties in cells administered exogenously into the intrasynovial space may be beneficial for intrasynovial tendon regeneration.

**Supplementary Information:**

The online version contains supplementary material available at 10.1186/s12917-021-02793-1.

## Background

Navicular disease is a common and longstanding cause of performance-limiting forelimb lameness in the horse [1]. With advances in diagnostic imaging, deep digital flexor tendon (DDFT) injury within the podotrochlear bursa is becoming among the most commonly diagnosed pathologies in navicular disease [2–4]. These diagnoses come with a poor prognosis for long-term athleticism and soundness [5–8]. Surgical [9], medical [2, 10], and conservative [2, 5, 10] treatments are undertaken to optimize tendon healing, but fibrotic healing often results with a high susceptibility to adhesion formation, recurrence and chronic lameness. In light of improved diagnostic capabilities and the rapidly expanding use of cell-based biologic therapies, there is potential for improved treatment outcomes in horses with navicular disease, but special consideration for its unique location and structures is warranted.

Intrasynovial DDFT contains superficial fibrocartilage as a functional adaptation to the compressive forces from the opposing navicular bone, which imparts a smooth surface and increased durability against abrasion [4, 11, 12]. The tendon’s cellular component, consisting of mature tenocytes and tendon-resident multipotent stem/progenitor cells is small (< 1%). However, it is responsible for tendon extracellular matrix (ECM) synthesis and turnover. In the fibrocartilaginous zone, ‘rounded/chondrocyte-like’ cells found within lacunae produce an amorphous ECM rich in proteoglycans [11, 13–16]. The fibrocartilaginous zone transitions to a tendinous zone composed of linear arrays of collagen fibers interspersed with spindle-shaped cells aligned along the longitudinal axis [11, 13–16], similar to cells of prototypical, extrasynovial tendon. Cells within the fibrocartilaginous and tendinous zones function to maintain the structural heterogeneity of intrasynovial tendon ECM that provides compressive stiffness as well as tensile strength, respectively.

Tendon stem/progenitor cells are thought to differentiate and play a crucial role in healing following injury [17]. Since their identification [17], tendon stem/progenitor cells have been gaining attention for potential healing attributes. They have been established from horses [18–20] and several other species [21–23]. In-vivo experimental studies have demonstrated that endogenous and exogenously administered tendon stem/progenitor cells participate in tendon homeostasis and repair [17, 24–28]. However, these studies are primarily focused on extrasynovial tendons. Further research is needed to identify and characterize intrasynovial fibrocartilaginous tendon-derived cells (TDC), like the DDFT within the podotrochlear bursa, to determine their potential role in tendon healing.

The purpose of this study was to isolate TDC separately from the fibrocartilaginous and tendinous zones of equine intrasynovial DDFT. Cells were isolated from forelimb DDFT within the proximal podotrochlear bursa and adjacent to the flexor surface of the distal sesamoid bone. Cells isolated from each zone were assessed for progenitor cell characteristics. First, we investigated the colony formation, plasticity during monolayer passage and immunophenotype characteristics (MSC surface markers, CD 90, CD 29 and CD 44) of the two TDC subpopulations. We then assessed the in vitro trilineage differentiation capacities of third passage TDC from the fibrocartilaginous (fTDC) and tendinous (tTDC) zones in relation to their respective terminally differentiated cells. Lastly, zonal variations in the aforementioned characteristics between fTDC and tTDC were evaluated.

## Results

### Morphology, clonogenicity and proliferation of intrasynovial TDC

**Fig. 1:**
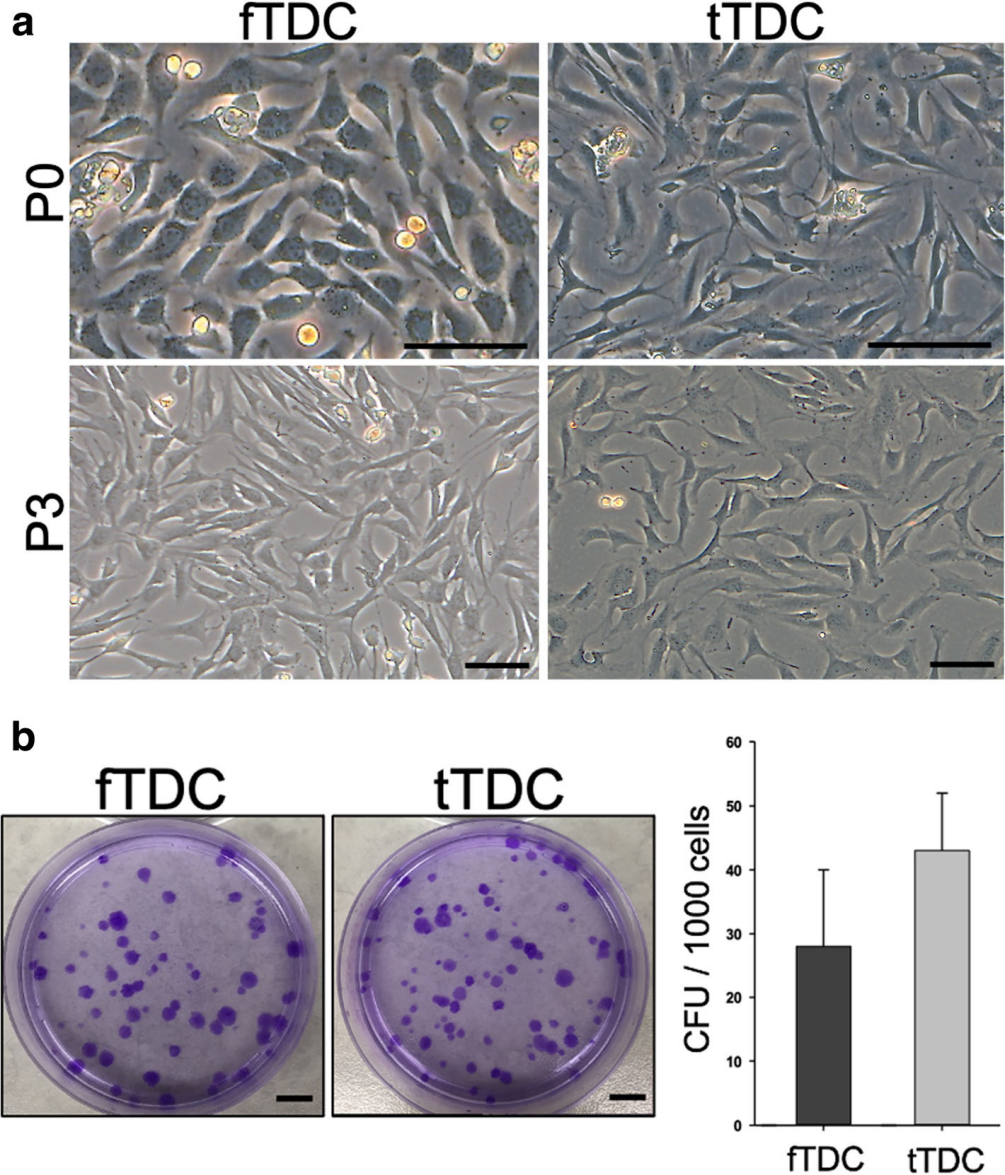
Equine intrasynovial TDC in vitro morphology and CFU. **a** Morphology of TDC during monolayer passage. At passage 0 (P0), attached cells from the fibrocartilaginous and tendinous zones were polygonal and elongate/spindle-shaped cells, respectively. At passage 3 (P3), both fTDC and tTDC were homogenous and exhibited fibroblast-like morphology. Scale bar = 100 microns. **b** Colony forming unit assay of cells from the fibrocartilaginous and tendinous zones after 10 days of culture. Scale bar = 1 cm. Bars represent mean ± SD CFU

At day 3 of culture, attached cells from the fibrocartilaginous zone exhibited polygonal morphology, and cells from the tendinous zone were elongate and spindle shaped. By the third passage, both fTDC and tTDC were homogenous and exhibited fibroblast-like morphology (Fig. 1a).

The clonogenic capacity of intrasynovial TDC was determined using the colony forming unit (CFU) assay. The colonies from both fibrocartilaginous and tendinous zones were heterogenous in size and cell density (Fig. 1b). After 12–14 days, cells from the fibrocartilaginous and tendinous zones formed 28 ± 12 and 43 ± 9 colonies (*P* = 0.12), respectively from 1000 plated cells (Fig. 1b).

The time from initial plating of enzyme-digested fibrocartilaginous and tendinous zone cells to 70–80% confluence was 10.2 ± 1.2 days and 9.1 ± 1.6 days (*P* = 0.6), respectively. Population doubling time and population doublings during passage 1 and 2 were not significantly different between zones (Table 1).

**Table 1:** Population doubling (PD) and Population doubling time (PDT) of cells from the fibrocartilaginous and tendinous zones during first (P1) and second passages (P2) of monolayer expansion (mean ± SD days; *n* = 3)

	P1 PD	P1 PDT	P2 PD	P2 PDT
Fibrocartilaginous zone	1.6 ± 0.3	2.1 ± 1.2	1.8 ± 0.5	2.4 ± 1.1
Tendinous zone	1.8 ± 0.5	2.3 ± 0.9	1.9 ± 0.2	2.9 ± 1.6
*p* value	0.32	0.71	0.2	0.15

### Phenotype characterization of fTDC and tTDC

**Fig. 2:**
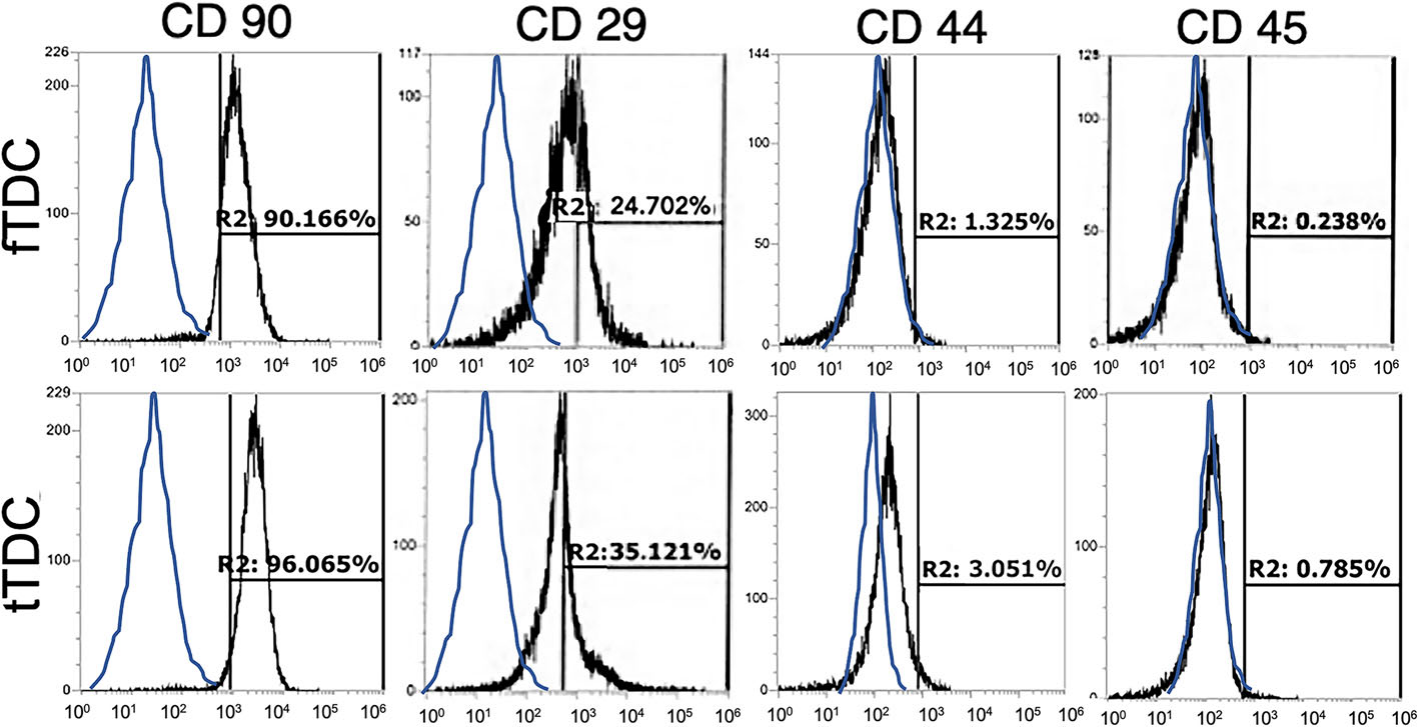
Equine intrasynovial TDC immunophenotype. Representative immunophenotype of third passage fTDC and tTDC. Black histograms indicate epitope-specific antibodies, while the blue histograms represent distributions of isotype IgG antibody (control)

MSC surface markers, CD 90, CD 29 and CD 44, as well as hematopoietic marker CD 45 were assessed. Monolayer passage enriched for CD 90^+^, CD 29^+^ cells in both fTDC and tTDC; the cells expressed low levels of CD 44 and were negative for the hematopoietic marker, CD 45 (Fig. 2). From the time of enzyme digestion to third passage, the percentage of CD 90^+^ cells increased from ~ 70% to 85–95%. Less than 5% of the cells were positive for CD 44. The rates of CD 90^+^, CD 29^+^, CD 44^+^ and CD 45^+^ cells did not significantly differ between fTDC and tTDC (Supplementary Table 1).

**Fig. 3:**
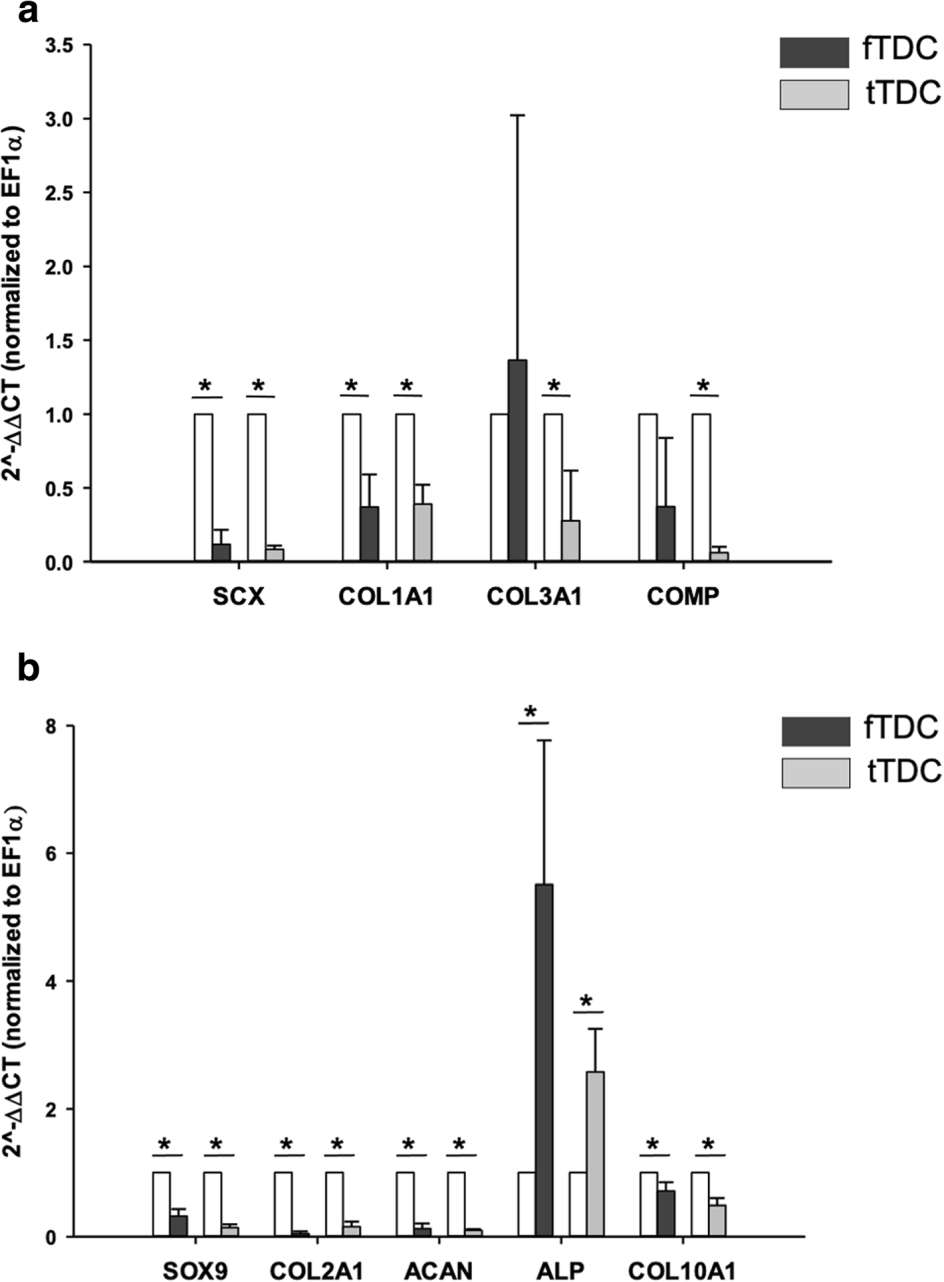
Equine intrasynovial TDC gene expression. Relative **a** tenogenic and **b** chondrogenic gene expressions of fTDC and tTDC. Bars represent mean ± SD. * represents significant difference (*P* ≤ 0.05) from respective terminally differentiated cells (unshaded bars). *n* = 3

Tenogenic (Fig. 3a), chondrogenic (Fig. 3b) and osteogenic mRNA of terminally differentiated cells, and fTDC and tTDC were assessed. Monolayer passage significantly down-regulated SCX (~ 10-fold) and COL1A1 (~ 3.5-fold) mRNA in fTDC and tTDC. There were no significant differences between the tenogenic mRNA expressions of fTDC and tTDC. SOX-9, COL2A1, ACAN and COL10A1 mRNA of fTDC and tTDC were significantly down-regulated relative to the respective terminally differentiated cells. In contrast, ALP mRNA of fTDC (5-fold; *P* = 0.026) and tTDC (2.8-fold; *P* = 0.015) was significantly increased. Similar to tenogenic mRNA, there were no significant differences between fTDC and tTDC chondrogenic mRNA. Osteogenic mRNA, RUNX2 and SPARC were < 2-fold increased (*P* > 0.05) from respective terminally differentiated cells.

### Chondrogenic differentiation

**Fig. 4:**
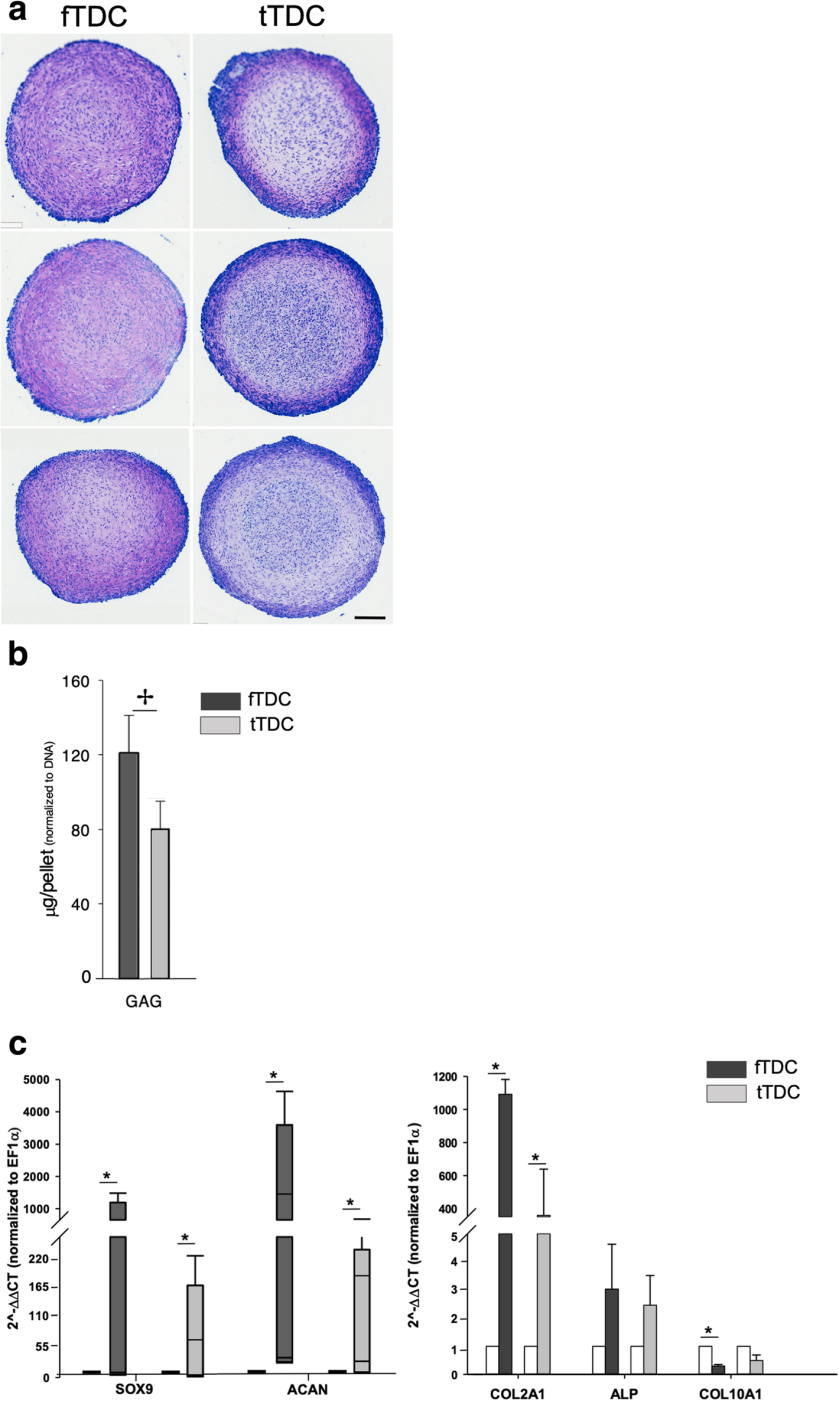
Chondrogenesis of fTDC and tTDC. a Toluidine blue staining of day 20 chondrogenic pellets. Stain uptake and metachromatic hue reflective of sGAG content are higher in fTDC pellets (Figure panels include three independent experiments). Scale bar = 100 microns. b sGAG contents of day 20 fTDC and tTDC pellets normalized to DNA content. c Relative chondrogenic gene expression. Box-whisker plots represent medians and inter-quartile ranges (SOX-9 and ACAN), and bars represent mean ± SD (COL2A1, ALP, COL10A1). * represents significant difference (*p* ≤ 0.05) from respective terminally differentiated cells (unshaded bars). ✢ represents significant difference (*p* ≤ 0.05) between day 21 fTDC and tTDC. *n* = 5

Toluidine blue staining intensity of day 20 fTDC pellets was higher and uniformly distributed throughout the pellet demonstrating increasing sulfated glycosaminoglycan (sGAG) content compared to tTDC pellets (Fig. 4a). Correspondingly, sGAG quantity of day 20 fTDC chondrogenic pellets was significantly (*P* = 0.02) increased from tTDC pellets (Fig. 4b).

Chondrogenic stimulation significantly upregulated day 20 fTDC and tTDC pellets’ SOX-9, ACAN and COL2A1 mRNA, compared to respective terminally differentiated cells (Fig. 4c). There were no significant differences between day 20 fTDC and tTDC values. Hypertrophic phenotype markers, ALP and COL10A1 were unaffected by chondrogenic stimulation.

### Osteogenic differentiation

**Fig. 5:**
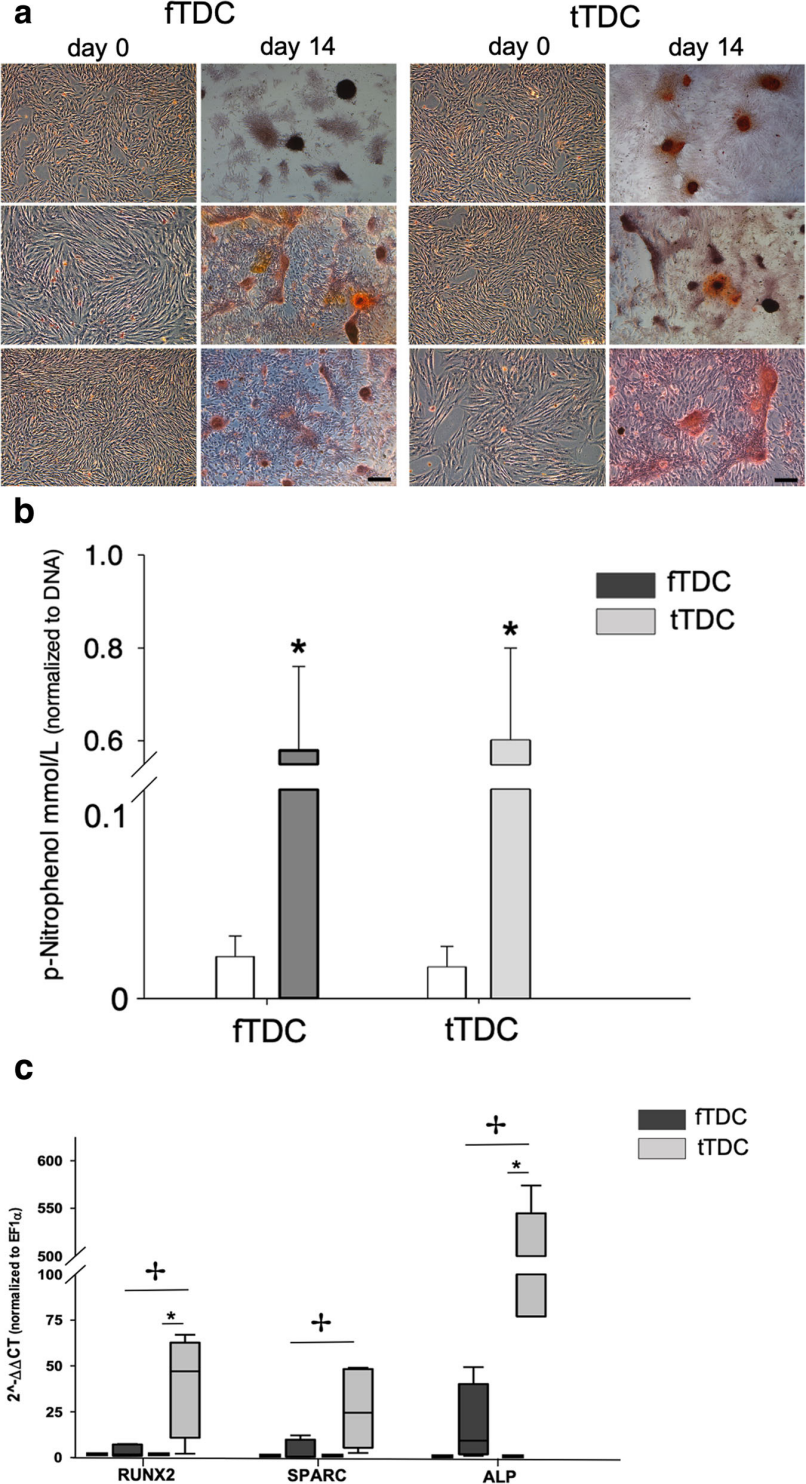
Osteogenesis of fTDC and tTDC. **a** Alizarin Red staining of day 0 and 21 osteogenic cultures (Figure panels include three independent experiments). Scale bar = 100 microns. **b** Alkaline phosphatase bioactivities of day 0 and 21 fTDC and tTDC osteogenic cultures normalized to DNA content. **c** Relative osteogenic gene expression. Box-whisker plots represent medians and inter-quartile ranges. * represents significant difference (*P* ≤ 0.05) from respective terminally differentiated cells. ✢ represents significant difference (*P* ≤ 0.05) between day 21 fTDC and tTDC. *n* = 5

Alizarin Red staining of day 21 osteogenic cultures demonstrated cell aggregation in both fTDC and tTDC; however minimal mineralized matrix secretion was present in fTDC and tTDC (Fig. 5a). Compared to day 0 cells, alkaline phosphatase bioactivities (normalized to DNA content) of day 21 fTDC (5.6-fold; *P* = 0.02) and tTDC (6-fold; *P* = 0.01) cultures were significantly increased (Fig. 5b). Day 21 ALP bioactivities of fTDC and tTDC were not significantly different (*P* = 0.07).

Day 21 fTDC RUNX2, SPARC and ALP mRNA were not upregulated compared to terminally differentiated cells. In contrast, day 21 tTDC RUNX2 and ALP mRNA were significantly increased. Day 21 tTDC osteogenic mRNA was significantly higher than the corresponding fTDC values (Fig. 5c).

### Adipogenic differentiation

**Fig. 6:**
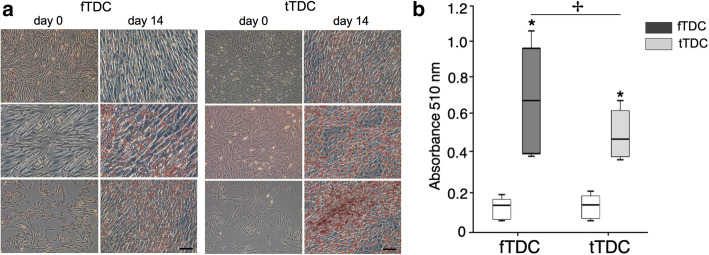
Adipogenesis of fTDC and tTDC. **a** Oil-Red-O staining of day 0 and 14 adipogenic cultures. Cytoplasmic lipid droplets were present in day 14 fTDC and tTDC; however, the cells retained their fibroblast morphology (Figure panels include three independent experiments). Scale bar = 100 microns **b** Absorbance readings (mean ± SD) following dye elution of Oil-Red-O-stained day 0 and 14 cultures. * represents significant difference (*P* ≤ 0.05) from day 0 cultures. ✢ represents significant difference (*P* ≤ 0.05) between day 14 fTDC and tTDC. *n* = 3

Day 14 Oil-Red-O stained adipogenic cultures showed cytoplasmic lipid droplet accumulation in both fTDC and tTDC, compared to day 0 cultures (Fig. 6a); however, stain uptake was minimal, and the cells retained their fibroblast morphology. Dye elution and quantification (Fig. 6b) demonstrated 3- to 5.8-fold (*P* = 0.03) and 2- to 3.6-fold (*P* = 0.02) increases in fTDC and tTDC, respectively from day 0 cultures. Absorbance readings of day 14 fTDC adipogenic culture was 1.3- to 1.9-fold increased from tTDC (*P* = 0.03).

## Discussion

Intrasynovial TDC evaluated in this study were isolated using low-density plating method commonly used to isolate tendon stem/progenitor cells from extrasynovial equine [18–20, 29], human [17], and mouse [17, 30] tendon. We have designated these cells as TDC since further characterization is needed to determine if they possess stem/progenitor cell properties or if they are differentiated cells. The clonogenic and monolayer passage characteristics of fTDC and tTDC were similar and consistent with existing extrasynovial tendon studies, including those performed in horses [17–20]. After monolayer passage fTDC and tTDC became homogenous, spindle-shaped cells. The tenogenic and chondrogenic markers in fTDC and tTDC significantly decreased relative to their respective terminally differentiated cells and were not significantly different between zones. This plasticity property is seen in other tissue-derived stem/progenitor cells and highlights the key role of the ECM on their in-situ phenotype and bioactivities [17, 31, 32].

There was no difference between fTDC and tTDC MSC surface marker profiles, and they are comparable to extrasynovial counterparts [17, 33] and bone marrow-derived MSC [18–20, 34] with exception of CD44. Less than 5% of the cells were positive for CD44, a mesenchymal stromal cell marker that has been identified in TDC from equine extrasynovial tendon [18], using equine-specific antibody as herein. Basal fTDC and tTDC surface marker phenotypes assessed were limited in this study, and more robust analyses of cell surface markers and gene expression representing stem/progenitor cells such as CD73, CD105, Oct-4 are warranted to determine the significance of the CD44^−^ cells. CD44 was not evaluated in other equine tendon stem/progenitor cell studies [19, 20].

Reflective of the in-situ chondrocyte-like morphology of tendon fibrocartilage cells, fTDC were largely restricted to chondrogenesis in vitro, whereas tTDC underwent osteogenic and chondrogenic differentiation. Although the basal (non-induced) osteogenic and chondrogenic mRNA profiles of fTDC and tTDC were not significantly different, basal fTDC SOX9 mRNA was 3-fold higher than tTDC and approached significance (*P* = 0.06; Fig. 4b). Subsequently, with chondrogenic stimulation, fTDC SOX-9 and ACAN mRNA expressions were 5- (*P* = 0.07) and 3.5- (*P* = 0.06) fold higher than tTDC. Toluidine blue stain uptake reflecting the sGAG content was also higher in fTDC pellets and was uniformly distributed throughout the pellet, whereas the stain uptake was just localized to the periphery of tTDC pellets (Fig. 4a) and corroborates with increased pellet GAG content in fTDC compared to tTDC. These findings suggest that fTDC may be ‘committed’ to a chondrogenic phenotype. Multi-assay panels for rigorous assessment of lineage commitment and alternative methods for isolation are required prior to drawing this conclusion. It also possible that the low-density plating method selects for a chondrogenic fTDC subpopulation. The minimal adipogenic capacity observed was in keeping with a previous report on equine superficial digital flexor TDC [18].

The common clonogenic, proliferative, and immunophenotype characteristics, and varying trilineage differentiation potentials of TDC isolated from morphologically distinct tendon zones are consistent with reports on meniscal [35] and intervertebral disc (IVD) [36] tissues. In vitro chondrogenic restriction of stem/progenitor cells isolated from the inner zone of meniscus and IVD nucleus pulposus is partially implicated in the limited intrinsic repair capacity of these tissue zones. Based on our results, further assessment is necessary to determine if the same is true of fibrocartilaginous tendon.

While we have described fTDC characteristics in relation to their terminally differentiated cell counter parts and tTDC, comparative analyses with matched ‘gold standard’ bone marrow-derived MSC and extrasynovial TDC could be more informative regarding their stem/progenitor cell characteristics. The following study factors are to be taken into consideration. Intrasynovial TDC were isolated from healthy donors of a wide age range, albeit representative of those susceptible to clinical disease [2–4, 11, 12, 37]. Secondly, low-density plating was used, but alternate approaches such as differential substrate adherence or cell surface epitope fluorescent activated cell sorting (FACS)-based separation could also be evaluated. However, feasibility of these technologies with equine cell stocks is limited. Lastly, trilineage differentiation mRNA expression in fTDC and tTDC was compared to the respective terminally differentiated cells. Maintaining terminally differentiated subpopulations in basal medium for the same duration as induction media would have facilitated spontaneous differentiation assessments.

The diminished healing capacity of intrasynovial tendons is attributed in part due to limited intrinsic healing mechanisms and inherent low tissue cellularity. The limitations in healing are also reflective of the mechanical environment within the tissue and persistent inflammation, consequently resulting in net tissue catabolism. Longer-term in vivo experimental studies based on the results of this study are warranted to delineate the mechanisms related to zonal healing differences and the potential role of TDC, and cell-based therapies in enhancing intrinsic repair. Promoting chondrogenic properties in cells administered exogenously into the intrasynovial space and in cells that are used to revitalize decellularized autografts may be beneficial for intrasynovial tendon regeneration.

## Conclusions

This study investigated the characteristics of TDC isolated from the fibrocartilaginous and tendinous zones of the equine intrasynovial DDFT. Both TDC subpopulations exhibited clonogenicity and expressed similar cell surface marker profiles. After 2 monolayer passages, chondrogenic and tenogenic markers were downregulated in fTDC and tTDC indicative of plasticity property. There were no significant differences in the basal tenogenic, osteogenic and chondrogenic marker expressions of fTDC and tTDC. Trilineage differentiation demonstrated that fTDC were largely restricted to a chondrogenic lineage, whereas tTDC underwent both osteogenic and chondrogenic differentiation. Both fTDC and tTDC displayed weak adipogenic differentiation potentials. These results provide a foundation for studies exploring cell-based therapies for intrasynovial tendon repair as these TDC are potential targets to enhance intrinsic repair capacity.

## Methods

### Intrasynovial TDC isolation and culture

**Fig. 7:**
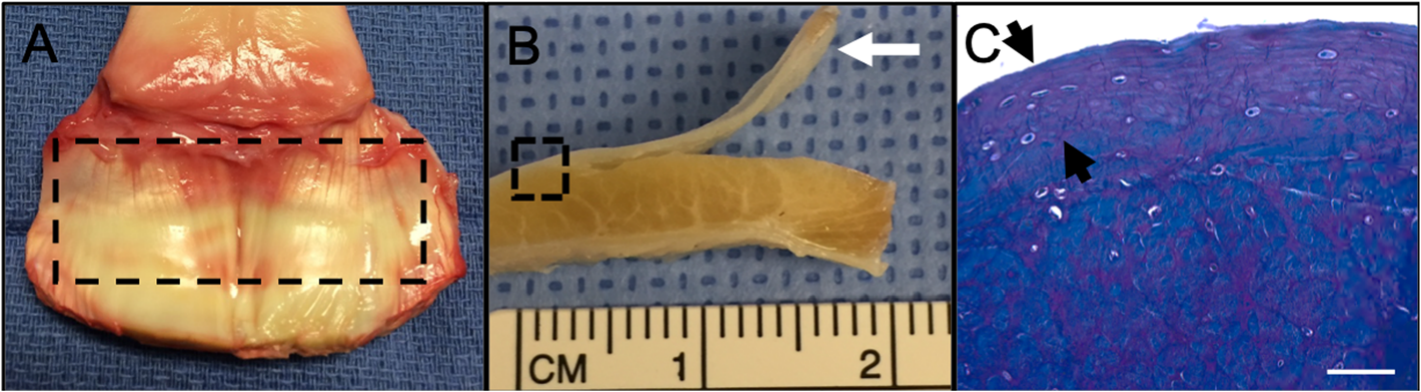
Equine intrasynovial DDFT structure and TDC isolation. **a** Equine forelimb intrasynovial DDFT within the hoof capsule. The outlined area (dashed line) represents tissue within the podotrochlear bursa that was used to isolate TDC. **b** Gross cross-section of DDFT with dorsal fibrocartilage dissected (arrow) from the underlying tendinous tissues. **c** Toluidine blue-stained cross section of DDFT (area outlined with a dashed line in (**b**) that depicts chondrocyte-like cells within the proteoglycan-rich dorsal fibrocartilage (black arrow heads). Scale bar = 500 microns

All procedures were approved by the University Institutional Animal Care and Use Committee. DDFT tissue was harvested from the forelimbs of five equine cadavers euthanized for reasons unrelated to musculoskeletal disorders (age range 6 to 12 years). Intrasynovial DDFT tissue within the proximal aspect of the podotrochlear bursa and adjacent to the distal sesamoid bone was used for TDC isolation and was determined free of gross pathology at the time of harvesting [11, 38]. The dorsal fibrocartilaginous and underlying tendinous zones of DDFT were dissected by gross assessment (Fig. 7). From each horse about 1.5 and 3 g of dorsal fibrocartilaginous and underlying tendinous tissues were obtained, respectively.

The tissue specimens were diced (0.25-cm^3^) and cellular fractions isolated by dissociation in Dulbecco’s modified Eagle’s medium (DMEM; Gibco®, Carlsbad, CA) containing collagenase (Worthington, Lakewood, NJ) and supplemented with 2% fetal bovine serum (Gibco®) and 1% penicillin-streptomycin (Gibco®) at 37 °C for 18 h as previously described [17–19]. Following optimization of tissue digestion; the fibrocartilaginous and tendinous zone tissues were digested with 0.15% collagenase type II and 0.2% collagenase type I, respectively. Primary cells from respective digests isolated by sequential filtration (40 μm) and centrifugation were seeded at 2000 cells/cm^2^ in monolayer cultures for expansion [18, 19, 30]. At day 3 after primary culture, the unattached cells were removed by washing with PBS. Fresh culture medium was added every 3 days. Once discernable colonies (> 50 cells) were observed in both TDC subpopulations, they were detached using 0.05% trypsin-EDTA (Gibco®) and sub-cultured. Subsequently, cells were detached at ~ 80% confluence and passaged twice. Third passage cells from the fibrocartilaginous and tendinous zones, designated as fTDC and tTDC, were used for immunophenotyping and trilineage differentiation assays.

### Colony-forming unit (CFU) assay and cell proliferation

Enzyme digested cells from fibrocartilaginous and tendinous zones were plated at 10 cells/cm^2^ for colony forming unit (CFU) assay [17]. After 14 days of culture, CFU assay was performed with 0.5% crystal violet staining after 4% paraformaldehyde fixation. All stained colonies (> 2 mm diameter) were manually counted.

Cell proliferation during first and second passages (P1 and P2) of TDC from both zones was calculated as population doublings using the formula: Log_2_ (harvested cell number/seeded cell number). Population doubling times for TDC during passage 1 and 2 were calculated by dividing the time of each passage by the corresponding population doubling value [18].

### Phenotype characterization of monolayer expanded TDC

Single-cell suspensions from TDC dissociated with 0.05% Trypsin-EDTA were used.

### FACS and MSC surface markers

One million terminally differentiated cell from each zone, as well as fTDC and tTDC, were blocked with 10% BSA suspended in FACS buffer (pH 7.4 PBS with 1% BSA) for 20 min prior to washing. Cells were washed in PBS and resuspended in fluorescent conjugated or unconjugated primary antibodies and incubated at 4 °C for 30 min. The following antibodies were used according to the manufacturers’ recommendations: anti-human conjugated anti-CD29-Alexa 488 (BioLegend, San Diego, CA); anti-horse conjugated anti-CD44-RPE (BioRad, Hercules, CA); anti-horse non-conjugated anti-CD90- conjugated with Alexa 647 (Accurate Chemicals, Westbury, NY) and anti-human conjugated anti-CD45-Alexa 488 (BioRad) [18]. Cells were washed and resuspended in FACS buffer and analyzed by flow cytometry (Attune NxT Fluocytometer, ThermoFisher Sci, Waltham, MA). Cells blocked with 10% BSA and without antibody incubation or in the presence of secondary antibody alone were used as controls. A threshold gating out at least 95.5% of control cells was used. The percentage of positive cells was calculated and expressed as that exceeding the threshold.

### Lineage-specific gene expression

Tenogenic, chondrogenic and osteogenic marker gene expressions were assessed in the respective terminally differentiated cells as well as fTDC and tTDC. Three million cells were stored for RNA isolation and quantitative real time-PCR (qRT-PCR) using SYBR Green Master Mix (Applied Biosystems, Foster City, CA), as detailed below for the following genes: scleraxis (SCX), tenomodulin (TNMD), collagen type I (COL1A1), collagen type III (COL3A1), cartilage oligomeric matrix protein (COMP); sex determining region Y-box 9 (SOX-9), collagen type II (COL2A1), aggrecan (ACAN), alkaline phosphatase (ALP), collagen type X (COL10A1); runt-related transcription factor 2 (RUNX2) and osteonectin (SPARC).

### Trilineage differentiation and phenotypic assays

Standard protocols for adipogenesis, osteogenesis and chondrogenesis assays were employed for trilineage differentiation assays of fTDC and tTDC, as described below [17–19].

### Chondrogenic culture

Pellet cultures were established in microcentrifuge tubes from fTDC and tTDC by resuspending 5 × 10^5^ cells/mL in chondrogenic medium (DMEM containing 100 U of sodium penicillin/mL and 100 μg of streptomycin sulfate/mL supplemented with 100 ηM dexamethasone, 25 μg/ml ascorbic acid, 10 ηg/ml TGF-β1, and 1% ITS media supplement), and pelleting 500 μl aliquots of the cell suspensions at 400 rcf for 8 min. Chondrogenic cultures were maintained for 20 days. Chondrogenic medium was replaced every 2 days.

Representative pellet sections were stained with toluidine blue to assess sGAG deposition after 20 days of chondrogenic culture. Cell pellets were fixed in 4% paraformaldehyde, dehydrated and embedded in paraffin. Six micron-thick sections were stained with toluidine blue prior to acquiring photomicrographs. Total sGAG within representative pellets (3 replicates each per horse) was measured using dimethyl methylene blue (DMMB) colorimetric assay [39]. Upregulation of chondrogenic genes SOX-9, COL2A1, ACAN, ALP and COL10A1 was assessed by qRT-PCR, as detailed below.

### Osteogenic culture

fTDC and tTDC were plated at 10,000 cells/cm^2^ in 6- and 12-well plates and cultured in complete DMEM until they reached 70–80% confluence. Complete DMEM was then substituted with osteogenic medium (DMEM containing 10% fetal bovine serum, 100 U of sodium penicillin/mL, and 100 μg of streptomycin sulfate/mL supplemented with 10 mM β glyceraldehyde-3-phosphate, 50 μg/mL ascorbic acid, 100 ηM dexamethasone). The medium was replaced every 2 days. The cultures were maintained for 21 days.

Alizarin Red staining was used to assess mineralized matrix deposition. The cell-matrix layer was washed with PBS and fixed with 70% ethanol and stained with 2% Alizarin Red stain for 10 mins. Photomicrographs were obtained prior to osteogenic differentiation and at day 21 of osteogenic culture. Alkaline phosphatase bioactivity of representative cultures was measured (LabAssay ALP, Wako Chemicals, Richmond, VA) and normalized to the total DNA content (Quant-iT Pico Green kit, Life Technologies, Waltham, MA) [40]. Upregulation of osteogenic transcription factor RUNX2 and osteoblast-specific proteins ALP and SPARC mRNA were assessed by qRT-PCR, as detailed below.

### Adipogenic culture

fTDC and tTDC were plated at 5000 cells/cm^2^ in 12-well plates and cultured in complete DMEM until they reached 80% confluence. Complete DMEM was then substituted with adipogenic medium (DMEM containing 10% rabbit serum, 100 U of sodium penicillin/mL and 100 μg of streptomycin sulfate/mL and supplemented with 1 mM dexamethasone, 100 mM indomethacin, 10 mg/mL insulin, and 500 mM isobutylmethylxanthine). Medium was replaced every 2 days. These cultures were maintained for 14 days.

Oil-Red-O staining of cultures was used to detect intracellular lipid accumulation. Cell monolayers were washed with PBS, fixed with 70% ethanol for 30 min, and stained with 0.3% Oil-Red-O stain for 1 h. Hematoxylin was added for 10 min. Photomicrographs were obtained prior to adipogenic differentiation and at day 14 of adipogenic culture. Subsequently, Oil-Red-O stain was eluted with 2-propanol and absorbance of the eluate was spectrophotometrically quantified (Microplate Reader, Tecan Group Ltd., Switzerland) at 510 nm [41].

#### RNA isolation and qRT-PCR

Total RNA was isolated using a previously described protocol [18]. The samples were homogenized in a guanidinium thiocyanate-phenol-chloroform solution reagent (TRIzol, ThermoFisher Scientific) according to manufacturer’s suggested protocol. RNA isolation from the chondrogenic pellets included the high-salt precipitation variation, to minimize co-precipitation of proteoglycans. The resultant pellet was purified using RNeasy silica columns that included on-column DNase digestion. One μg of RNA from each sample was reverse-transcribed (Superscript IV, ThermoFisher Sci) using oligo (dT) primers. Equine gene-specific primers were designed from published sequences in Genbank and using ClustalW multiple sequence alignment (available at www.ebi.ac.uk) (Table 2). Primer specificity was confirmed by cloning and sequencing the amplicons during optimization experiments, as previously described [18, 24, 42]. PCR amplifications were catalyzed by Taq DNA polymerase (QuantStudio 3, Applied Biosystems, ThermoFisher Sci) in the presence of Sybr Green. Gene expression was quantified using the 2^-∆∆CT^ method, normalized to expression of housekeeping gene, elongation factor-1α (EF1α) [43].

**Table 2:** Primer sequences used for qRT-PCR

Gene		Sequence	Amplicon (bp)
SCX	S	5′ GAC CGC ACC AAC AGT GTG AA	231
	A	5′ TGG TTG CTG AGG CAG AAG GT	
COL1A1	S	5′ GAA AAC ATC CCA GCC AAG AA	231
	A	5′ GAT TGC CAG TCT CCT CAT CC	
COL3A1	S	5′ AGG GGA CCT GGT TAC TGC TT	215
	A	5′ TCT CTG GGT TGG GAC AGT CT	
COMP	S	5′ TCA TGT GGA AGC AGA TGG AG	223
	A	5′ TAG GAA CCA GCG GTA GGA TG	
RUNX2	S	5′ CAG ACC AGC AGC ACT CCA TA	177
	A	5′ CAG CGT CAA CAC CAT CAT TC	
SPARC	S	5′ AAC CTT CTG ACC GAG AAG CA	190
	A	5′ TGG GAC AGG TAC CCA TCA AT	
ALP	S	5′ TGG GGT GAA GGC TAA TGA GG	221
	A	5′ GGC ATC TCG TTG TCC GAG TA	
SOX-9	S	5′ GAA CGC ACA TCA AGA CGG AG	304
	A	5′ CTG GTG GTC TGT GTA GTC GT	
COL2A1	S	5′ AGC AGG AAT TTG GTG TGG AC	223
	A	5′ TCT GCC CAG TTC AGG TCT CT	
ACAN	S	5′ GAC GCC GAG AGC AGG TGT	202
	A	5′ AAG AAG TTG TCG GGC TGG TT	
COL10A1	S	5′ TGC CAA CCA GGG TGT AAC AG	244
	A	5′ ACA TTA CTG GGG TGC CGT TC	
EF1α	S	5′ CCC GGA CAC AGA GAC TTC AT	328
	A	5′ AGC ATG TTG TCA CCA TTC CA	

### Statistical analysis

Normal distribution of quantitative data was assessed using the Kolmogorov-Smirnov test. Data are expressed as mean ± standard deviation or as median and interquartile ranges. Comparative differences between cells from fibrocartilaginous and tendinous zones were analyzed for CFU and cell proliferation. Plasticity during monolayer passage with respect to cell surface antigens and tenogenic, osteogenic and chondrogenic marker gene expressions (between terminally differentiated cells and, fTDC and tTDC) was evaluated. Subsequently, trilineage differentiation potentials of fTDC and tTDC were assessed via fold change in gene expression of end-point induction cultures from respective terminally differentiated cells. One-way analysis of variance (ANOVA) or its non-parametric equivalent, Kruskal Wallis test was used to analyze and compare results within and between cells from fibrocartilaginous and tendinous zones. All analyses were conducted using SigmaStat 4 software (Systat Software, San Jose, CA). Significance was set at *P* ≤ 0.05.

## Supplementary Information

**Additional file 1: Supplementary Table 1.** Percentage positive cells recorded with fluorescent activated cell sorting (FACS) for MSC surface markers (CD 90, CD 29 and CD 44) and hematopoietic marker, CD 44 of third passage fTDC and tTDC.

## Data Availability

The datasets used and/or analyzed during the current study are available from the corresponding author on reasonable request.

## Abbreviations

DDFT: Deep digital flexor tendon
ECM: Extracellular matrix
TDC: Tendon-derived cells
MSC: Mesenchymal stem cell
fTDC: TDC from fibrocartilaginous zone
tTDC: TDC from tendinous zone
CFU: Colony forming unit
FACS: Fluorescent activated cell sorting
SCX: Scleraxis
COL1A1: Collagen type I
SOX-9: Sex determining region Y-box 9
COL2A1: Collagen type II
COL3A1: Collagen type III
COMP: Cartilage oligomeric matrix protein
ACAN: Aggrecan
COL10A1: Collagen type X
ALP: Alkaline phosphatase
RUNX2: Runt-related transcription factor 2
SPARC: Osteonectin
EF1α: Elongation factor-1α
sGAG: Sulfated glycosaminoglycan
DMEM: Dulbecco’s modified Eagle’s medium
PBS: Phosphate buffered saline
DMMB: Dimethyl methylene blue
qRT-PCR: Quantitative real time polymerase chain reaction
ANOVA: Analysis of variance
