# Supplementary material for: Zonal characterization and differential trilineage potentials of equine intrasynovial deep digital flexor tendon-derived cells
Source: BMC Vet Res. 2021 Apr 1;17:138. doi: 10.1186/s12917-021-02793-1 (PMC8015054; doi:10.1186/s12917-021-02793-1)
Supplement: Supplementary file 1 — Additional file 1: Supplementary Table 1. Percentage positive cells recorded with fluorescent activated cell sorting (FACS) for MSC surface markers (CD 90, CD 29 and CD 44) and hematopoietic marker, CD 44 of third passage fTDC and tTDC. [file 12917_2021_2793_MOESM1_ESM.docx]

|  | CD 90 (%) | CD 29 (%) | CD 44 (%) | CD 45 (%) |
| --- | --- | --- | --- | --- |
| fTDC  (n=3) | 89.341 | 24.135 | 2.124 | 0.231 |
|  | 91.125 | 25.324 | 1.611 | 0.178 |
|  | 90.166 | 24.702 | 1.345 | 0.238 |
| Mean + SD (%) | 90.21 + 0.89 | 24.72 + 0.59 | 1.69 + 0.39 | 0.21 + 0.32 |
| tTDC  (n=3) | 95.435 | 34.124 | 3.210 | 0.612 |
|  | 95.122 | 35.656 | 3.106 | 0.766 |
|  | 96.065 | 35.121 | 3.051 | 0.785 |
| Mean + SD (%) | 95.54 + 0.48 | 34.96 + 0.77 | 3.12 + 0.08 | 0.72 + 0.09 |
| *P* Value | 0.3 | 0.09 | 0.1 | 0.12 |

Supplementary Table 1. Percentage positive cells recorded with fluorescent activated cell sorting (FACS) for MSC surface markers (CD 90, CD 29 and CD 44) and hematopoietic marker, CD 44 of third passage fTDC and tTDC.
